# Evaluation of Vaccine Immunogenicity—Correlates to Real-World Protection: Influenza

**DOI:** 10.3390/v16030441

**Published:** 2024-03-12

**Authors:** Csaba Laszlofy, Gyorgy Fazekas, Zoltan Barath, Zoltan Vajo

**Affiliations:** 1 Department of Prosthodontics, Faculty of Dentistry, University of Szeged, 6720 Szeged, Hungary; dr.csaba.laszlofy@sanoral.com (C.L.); barath.zoltan@stoma.u-szeged.hu (Z.B.); 2 Fluart Innovations Ltd., 2097 Pilisborosjeno, Hungary; gyorgy.fazekas@fluart.hu; 3 Department of Family Medicine, Semmelweis University Medical School, 1085 Budapest, Hungary

**Keywords:** influenza, vaccine, immunity, antibody, lymphocyte

## Abstract

Recent events highlighted that, despite decades of studying vaccine immunogenicity and efforts toward finding correlates of protection, evaluating real-world vaccine efficacy as well as establishing meaningful licensing criteria still represents a significant challenge. In this paper, we review all aspects of influenza vaccine immunogenicity, including animal and human challenge studies, humoral and cellular immunity parameters, and their potential correlation with real-life protection from disease.

## 1. Introduction

The evaluation of vaccine immunogenicity is of obvious importance, although it can be challenging, despite decades of clinical and laboratory studies. Finding surrogate markers of immunogenicity that correlate well with real-life protection is often difficult. We believed for decades that influenza vaccines were among the best studied, with well-established immunogenicity guidelines for licensing based on hemagglutinin inhibition (HI) assays [1,2]. More recently, besides hemagglutinin, recent studies have also focused on the activity of neuraminidase (NA) and cellular immunity induced by vaccines, and their possible correlation with protection [3,4,5,6].

After decades of using the same established immunogenicity parameters, based on cumulating new evidence, the European Union regulatory agencies have changed the previously published licensing criteria [7]. The most recent European guideline reflects a shift in the approach to the assessment of influenza vaccines, resulting in the removal of the previously recognized immunogenicity criteria, essentially based on HI assays, which have been the mainstay for licensing influenza vaccines for many years [8].

Below, we review all aspects of influenza vaccine immunogenicity, including animal and human challenge studies, humoral and cellular immunity parameters, and their correlation with real-life protection.

## 2. Human Challenge Studies

Although in vivo animal model systems have been widely used for the evaluation of the immunogenicity of influenza vaccines, the most direct information about vaccine efficacy can be gathered by utilizing human challenge studies. Results obtained by such systems describe the human immune responses and protection induced by influenza vaccines more directly, and parameters obtained by human challenge studies fit better to immune parameters compared to those obtained from animal challenge tests [9]. Moreover, human challenge systems can also be useful in studying the pathogenesis and immunology of the influenza infection itself.

Early studies of influenza infection, including human challenge studies following vaccination, suggested that HI antibody titers ranging from 1:15 to 1:65 may be associated with protection from illness in approximately 50% of subjects, and protection from illness is increased with higher titers [10,11]. Thus, early influenza vaccine guidelines were essentially based on HI titers of 1:40. Seroconversion, seroprotection, and Geometric Mean Titers (GMTs) were used as measures of vaccine immunogenicity and criteria for licensing [1,2]. As most such studies were conducted in healthy adults, the reliability of the 1:40 HI titer has been questioned, especially in elderly or pediatric patients, as well as individuals with comorbidities [12,13]. At least in the case of adjuvanted subunit vaccines in children, HI titers as high as 1:110 are needed to correlate with the conventional 50% protection rate [13].

As for the pathogenesis of natural influenza infection, as well as the effects of neuraminidase inhibitors, Hayden et al. examined several cytokines in human challenge studies and concluded that IL-6, TNF-α, and IFN-γ all correlate with the clinical and virological features of the illness [14]. In addition to establishing licensing criteria for seasonal vaccines and the pathogenesis of influenza infection, human challenge models may also be useful for the examination and characterization of a newly developed pandemic or even universal influenza vaccines [15].

In general, a wider spectrum of the utilization of human challenge models is still urgently needed in the context of examining new vaccine types (universal vaccines, cell culture-based vaccines) and more precise efficacy characterizations, as well as to optimize, standardize, and develop new influenza virus challenge strains, infection methods, and disease models [9,15]. In addition, the concept of immunological imprinting, or original antigenic sin, which was first described in the context of influenza viruses decades ago while studying the immune responses to infection by different influenza virus strains, should also be considered: it has been found that previous exposure to influenza virus strains could influence the immune response to subsequent exposure to other strains, either by vaccination or infection. According to such trials, antibody titers are the highest against influenza strains encountered during childhood [16]. Thus, with any human challenge or immunogenicity study, it is important to know and consider the previous vaccination and infection history of the participants. The limitations of human challenge studies are the obvious ethical concerns, compliance, and the difficulties with recruiting suitable volunteers.

## 3. Animal Challenge Models

Animal models have been utilized to study all aspects of influenza for decades. They helped with the initial isolation of the virus and its subsequent propagation. A well-established animal model is crucial to evaluate viral pathogenicity, transmission, and vaccine immunogenicity, as well as efficacy. Animal models are also used for the preclinical evaluation of candidate vaccines and for the development of entirely new types of antiviral agents. The most commonly used animal models are mice and ferrets, while some less frequently utilized experimental models include guinea pigs, swine, non-human primates, and various birds [17,18]. There are multiple deciding factors as to which particular experimental animal model should be used for any given task, such as the cost, ease of animal handling, receptor abundance and conformation, susceptibility to influenza virus strains, and obtaining supplies.

### 3.1. Mice

The benefits of the mouse model in clinical studies focusing on symptomatology are somewhat limited due to the lack of clinical symptoms following influenza infection in these animals. Mice usually do not develop fever, sneezing, and rhinorrhea following influenza infection, while some strains do cause clinical symptoms, but these may differ from clinical symptoms in humans. However, this model remains useful for serological studies after vaccination, offering the advantages of low cost, easy handling, and readily available supply chains. Hence, the mouse model has been the main staple animal model for examining the immunogenicity and efficacy of influenza vaccines for decades. One undeniable aspect of the mouse model is the accessibility of inbred mouse strains, each one with a distinctive genetic makeup and possibility of variable response to influenza viral infection. Animal treatment involves the injection of the given influenza vaccine and subsequent examination of the immune response by collecting the sera, lungs, lymph nodes, and spleen of the treated animals. Evaluation of humoral or cellular immune response is performed by the determination of induction of adaptive immune response. The most common laboratory methods for measuring the antibody-dependent response are ELISA, HI, or microneutralization assay (MN) [17].

As influenza viruses are not natural pathogens of mice, they have to be adapted for mouse models. This means that virus strains must be inoculated into mouse strains and passaged several times until the virus becomes pathogenic for mice. This procedure can be laborious and time-consuming [17]. Therefore, humanized mouse models have been developed, and used for the detection of cellular immunity, i.e., the recruitment of influenza antigen-specific CD8+ T-cells. Yu et al. developed a humanized mouse model by using ^137^Cs gamma irradiation and CD34+ hematopoietic stem cell inoculation [19]. This mouse humanized mouse challenge model was successfully used to study cellular immune response to influenza vaccination.

In addition, Song et al. developed a novel influenza mouse challenge model to mimic viral doses transmitted during natural infections of humans more precisely. In their model, instead of a one-time, high-dose infection, the authors used repeated low dose viral challenges. This novel system resulted in more severe disease with earlier morbidity and mortality, and the influenza vaccines tested protected mice from a high-dose challenge, but not from repeated low-dose challenges. The inflammatory response was also markedly higher in the several-dose infected mice. These results indicate a novel type of influenza animal challenge model, which might provide a more precise model for the evaluation of vaccine induced immunity [20].

### 3.2. Ferrets

Ferrets have been and remain one of the best animal models of influenza infection for multiple reasons, including that, unlike mice, they exhibit most of the clinical symptoms of influenza seen in humans, can be infected with human influenza virus strains without prior adaptation of the virus, and have the ability to transmit the influenza virus efficiently from animal to animal. This susceptibility of ferrets to human influenza viruses is due to the presence of α2-6-linked terminal N-acetylneuraminic sialic acids in their airways, which enables the binding and replication of the influenza virus [18].

On the other hand, the use of ferrets for animal models is limited by animal availability, high cost, and difficult handling. Nonetheless, the utilization of the ferret model in influenza vaccine research has grown extensively during the last decade, and become part of regulatory requirements [7,18]. In addition to their utilization in influenza vaccine studies, ferret models are also suitable to aid the development and evaluation of novel antiviral agents.

### 3.3. Avian Models

The avian immune system operates on the same general principles as the mammalian immune system. Antigenic stimulation initiates an immune response that involves cellular cooperation, most notably between macrophages, B-lymphocytes and T-lymphocytes. Influenza A is originally an avian virus and, thus, as far as animal models are concerned, using birds to evaluate vaccine effectiveness is theoretically the most logical method [21]. Even though the EMEA currently also recommends using ferrets for this purpose [4], for instance, highly pathogenic avian influenza virus (HPAI) strains cause significant mortality in unvaccinated chickens, but their virulence in mammals shows a great degree of variability. Such observations indicate that results obtained in HPAI mammalian protection models can vary significantly with the animal species and the dose and type of the experimentally applied challenge virus. It has previously been demonstrated that birds, especially chickens, Japanese quails (Coturnix-japonica), and Chinese painted quail (Coturnix-chinensis) were all useful animal models for testing protection against HPAI [21,22]. Chinese painted quails might be a more suitable model than chickens and Japanese quails in terms of their ease of general care, robustness, reproductive rate, and cost of maintenance [21]. The latter model also showed usefulness with serological testing in terms of HI and microneutralization assays, and thus, allowing the assessment of the correlation of immunological parameters with protection after vaccination [21]. Avian models are currently used primarily to test veterinary vaccines against highly pathogenic avian influenza virus strains.

Nonetheless, ideal animal challenge model systems that met the requirements of high susceptibility to a great variety of influenza viruses, easy animal handling, and fast, cost-effective, and reliable results are still needed.

## 4. Serological Testing

### 4.1. Hemagglutination Inhibition (HI)

Determining serum antihemagglutinin antibody titers for the studied influenza virus strain has been the gold standard for assessing the immunogenicity of influenza vaccines for many decades. The HI test is based on the inhibition of the interaction between the viral hemagglutinin (HA) glycoprotein and the sialic acid receptors of the surface of red blood cells by antibodies that are directed against the HA receptor binding pocket [23]. This test is a simple and inexpensive technique utilizing standard laboratory equipment and can be used for the identification of influenza virus subtypes, as well as for measuring HA-specific antibodies to the virus. This assay indirectly measures the ability of sera to disrupt the binding of HA to sialic acids on red blood cells, which serves as a correlate for the ability of these antibodies to prevent attachment to sialic acids on target cells in the respiratory tract [24]. Routinely, chicken red blood cells are used for this assay, although depending on the strain and subtype, erythrocytes from other species, such as turkeys, guinea pigs, pigeons, and horses, have all been utilized for HI testing [24,25].

Unfortunately, considerable variability can be introduced into the laboratory assay used to measure HI antibodies as a result of a number of factors including differences in viral strains, and the presence of non-specific inhibitors in the assay medium, and inconsistencies between erythrocyte batches from the same species. Using the readily available and widely utilized chicken erythrocytes, a considerable inter-, and sometimes even intralaboratory variability has been found. Studies have shown that HI titers reported for identical specimens in different laboratories can vary several by fold [24].

Thus, substantial efforts have been made to overcome the variability and improve the reliability of the HI assay for studying influenza vaccine immunogenicity and efficacy, such as the FLUCOP and CONSISE initiatives. FLUCOP is a large consortium of 22 members from eight European countries, including academic institutions, vaccine manufacturers, and public health authorities, supported by the Innovative Medicines Initiative Joint Undertaking. The FLUCOP project aims to build up a toolbox of standardized methods to smooth the progress of the development of existing and novel influenza vaccines [25]. Instead of using the widely utilized chicken erythrocytes, the FLUCOP standard recommends the use of turkey red blood cells and common reagents, and was established by the assimilation of numerous protocols from FLUCOP partners, using a large panel of sera and multiple influenza strains [25].

Therefore, appropriate controls and assay validation are crucial for the proper interpretation of HI antibody test results. It is also advisable that sufficient serum sample volumes should be obtained and stored for potential subsequent use in confirmatory or comparative assay studies if needed [25].

### 4.2. Chicken vs. Horse Red Blood Cells, the Variability of HI during Pandemics—Highly Pathogenic Avian Influenza (HPAI)

As discussed above, the HI test, in general, is a cost-effective and simple method used to assess immune responses to human influenza hemagglutinin. However, HI tests using the generally utilized chicken erythrocytes have been found to be insensitive to the detection of antibody responses to avian influenza hemagglutinin after vaccination or natural infection. This is due to the fact that avian influenza viruses preferentially bind to sialic acid receptors that contain N-acetylneuraminic acid alpha2,3-galactose (alpha2,3Gal) linkages, while human influenza virus strains preferentially bind to those containing N-acetylneuraminic acid alpha2,6-galactose (alpha2,6Gal) linkages.

Using horse erythrocytes in the HI test, and thereby increasing the proportion of alpha2,3Gal linkages available for binding, demonstrated improved detection of antibody to avian influenza H5 in human sera following vaccination with MF59-adjuvanted A/Duck/Singapore/97 surface antigen vaccine. The finding that the sensitivity of the HI test for the detection of antibodies against avian influenza viruses in human sera can be improved by replacing avian red blood cells with equine erythrocytes within the test has been proven multiple times [26,27]. The need for such improvement became clearly evident during the early 2000s when experts from the World Health Organization considered the HPAI strain influenza A(H5N1) to represent a pandemic alert level of stage 3 [28].

### 4.3. Changes in the Diagnostic of Human H3N2 Influenza Strains—Guinea Pig Erythrocytes

Human influenza virus strains have the ability to change rapidly, both genetically and immunogenically, and evolve into variants, and this is especially concerning for the A H3N2 strains. Thus, evaluation of the emerging H3N2 variants has become more complex over the past decades. Recently, many newly developed H3N2 strains appeared with highly altered HI activity. In the late 1990s, due to changes in their HA, H3N2 viruses started to lose their ability to agglutinate chicken red blood cells. It has even been found that circulating HA-deficient (i.e., with undetectable hemagglutinating activity against the conventionally used avian erythrocytes) H3N2 virus strains were present from 1999 to 2012 in the Netherlands [29].

It has been shown more recently that some H3N2 virus strains are not able to agglutinate avian red blood cells completely. However, these strains effectively agglutinate human and guinea pig erythrocytes. This suggests that these newly evolved virus strains favorably bind to α2,6 linked sialic acid molecules. Thus, the use of guinea pig red blood cells for HI is becoming more accepted in the case of H3N2 virus tests [30] Alternatively, in case of non-hemagglutinating influenza A virus strains, the checking of vaccine immunogenicity can be performed by ELISA and virus neutralization techniques instead of HI [31,32].

### 4.4. Neutralization

While HI tests detect antibodies that inhibit viral hemagglutination, the virus neutralization (VN) test, which is commonly used in a micro-neutralization (MN) format, detects antibodies that neutralize the virus and prevent its replication in living cells [32]. Not all antibodies that inhibit hemagglutination necessarily neutralize the virus, and on the contrary, not all virus-neutralizing antibodies can inhibit hemagglutination caused by the virus [33].

Generally, the micro-neutralization assay has been considered a more direct detection method of vaccine efficacy, because it does not measure the total hemagglutinin-specific antibodies, but instead directly detects the virus-neutralizing antibodies, which contribute to the inactivation of the haemagglutinin and so directly participate in the blocking of influenza virus proliferation. However, the MN assay, compared to HI, is more labor-intensive, time-consuming, and needs a living influenza virus and laboratory environment suitable for virus propagation (in the case of HPAI viruses, a Biosafety Level 3 facility). The evaluation of the test is performed by Enzyme-linked immunosorbent assays (ELISA) test. ELISA has been utilized to measure the absolute number of antibodies that can recognize an antigenic target such as whole influenza virus or purified HA [34]. The emergence of HPAI highlighted that MN has benefits for detecting antibodies to new influenza virus strains, since an infectious virus is used, and thus, the assay can be developed rapidly upon recognizing a novel variant, and it can be available before suitable recombinant or purified viral proteins become widely accessible for other assays.

Although MN has been in use for many years, the lack of data defining a 50% protective titer against influenza, as exists for HI, is a barrier to utilizing it formally as a correlate of protection [12]. In efforts to standardize MN and other serological assays for influenza, the Consortium for the Standardization of Influenza Seroepidemiology (CONSISE) was formed in 2011 by the WHO, CDC, and other participating organizations [35].

### 4.5. Single Radial Hemolysis (SRH)

The single radial hemolysis assay has been widely used since the 1970s to detect antibodies against several viruses (mainly hemagglutinating viruses), such as influenza, rubella, dengue and Japanese encephalitis [36]. Concerning influenza viruses, the method is used for the detection of serum antibodies against influenza virus hemagglutinin.

The assay itself is based on the passive hemolysis of red blood cells, mediated by complement components and induced by the antibody–antigen complexes, and it produces readily measurable areas of hemolysis, which, in turn, are proportionate to the concentration of anti-influenza antibodies present in the serum samples.

SRH is typically performed in agarose gels poured on glass plates. Influenza virus antigen-coupled erythrocytes (turkey, chicken) and complement (guinea pig) components are uniformly dispersed in the gel. The tested serum samples, which contain virus-specific antibodies, are filled into the holes punched into the gel. Antibodies in the tested serum samples diffuse in the gel radially and form immunocomplexes with the virus antigen-coupled erythrocytes. In the presence of complement, this immunocomplex formation results in spontaneous erythrocyte lysis. The size of the appearing rings is directly proportional to the concentration of virus-specific antibodies present in the sample [36,37,38].

Although the SRH assay has many advantages, such as being rapid, simple, reliable, reproducible, suitable for testing a large number of samples, and widely used for the evaluation of vaccine immunogenicity, unfortunately, the SRH assay has not yet been fully standardized [36]. In efforts to do so, Dominich et al. found that a ≥25 mm^2^ SRH output lysis zone corresponds well with an HI titer of ≥1:40, and thus, likely to ensure 50% protection [39]. Based on that, in addition to HI, the SRH assay has also been accepted as a correlate of protection by EMA guidelines [7].

Furthermore, Trombetta et al. concluded that the SRH assay is not affected by the subjectivity of the operator, which is a serious problem with HI, and that SRH is a robust and specific method for the detection of strain-specific antibodies against influenza virus strains [36].

In addition to studying vaccine immunogenicity, SRH is also used to verify influenza vaccine composition. The hemagglutinin content of the vaccines can be measured by a single radial immunodiffusion test, using reagents developed by the National Institute for Biological Standards and Control (NIBSC), United Kingdom [40]. It was concluded by Wood et al. that single radial diffusion may be of value for assays of the hemagglutinin concentration of both whole virus and subunit vaccines [40].

### 4.6. Neuraminidase Inhibition

Besides antibodies toward the HA protein, antibodies to alternative antigens of influenza viruses can also be measured. Neuramidase (NA) is a viral protein, which is present on the surface of influenza virus particles in large amounts. It is the second major surface protein after hemagglutinin, and thus, it plays an important role in the development of immunogenicity of influenza vaccines [41]. Inactivated whole virion, split and subunit influenza vaccines, as well as live attenuated influenza virus vaccines, all contain neuraminidase, but not in standardized amounts, while the currently licensed recombinant protein influenza vaccines and the presently developmental mRNA influenza vaccines do not contain any NA [42,43]. Anti-neuraminidase antibodies can be detected by ELISA, and antibodies functionally capable of disrupting NA activity can be detected through neuraminidase inhibition (NI) assays. It has been suggested that NI can be an important correlate of protection, independent of HI, particularly in the setting of secondary bacterial infections [41]. Thus, immunity against the NA component of the virion is desirable and should be considered in future influenza vaccines, as opposed to focusing on HA exclusively [44].

Since there is at least some evidence that NI titers are a correlate of protection against influenza virus-induced disease that is independent of HA-based immunity, a focus group on studying neuraminidase, NAction!, was started at a Centers of Excellence for Influenza Research and Surveillance meeting at the US National Institutes of Health to encourage research that helps to better comprehend neuraminidase-based immunity and how it can add to the design of improved and more broadly protective influenza vaccines [42].

## 5. Cellular Immunity

Influenza vaccine efficacy does not always correlate well with humoral immune responses. Recent reports indicate that the cellular immune response also contributes to protection; however, robust assays that correlate well with protection from disease are lacking at this point.

The detection of cellular immunity and the contribution of cellular components such as cytotoxic T-cells in vaccine-induced responses are extremely important in the case of new types of influenza vaccines, such as mRNA or DNA-based influenza vaccines. In contrast to the current vaccine technologies, such as inactivated whole virus, subunit, or split vaccines, which elicit mostly humoral immune responses, these new types of vaccines may induce broad humoral and cellular responses, through which they can produce strong protection [43,45]. Current influenza vaccine technologies mostly elicit humoral immune responses that block viral entry. On the other hand, mRNA vaccines may bring about better T-cell responses [43]. These cellular responses can be measured by measuring cytotoxic T-lymphocyte response, or cytokine production, such as IL4 and IFNg [45].

For licensing or postmarketing studies, the measurement of cell-mediated immunity (CMI) is strongly encouraged, at least in randomly selected subsets across the whole intended age range for vaccination. An evaluation of CMI may be particularly informative in elderly (e.g., aged 75 years and older) individuals, due to the recognized effects of immunosenescence and observations that antibody titers as measured by HI and VN may not reliably predict protection from developing disease in this age group [46]. Therefore, it is recommended that studies should monitor the quantity and quality of T-cell responses. Antigen-specific T-cell frequencies can be estimated, including Th1, Th2, T regulator cells, memory T-cells, and relevant cytokines. Additionally, a careful analysis of CD4+ and CD8+ responses, as well as the activation of memory B cells, would enable a more precise description of the effect of vaccination on antibody responses and clinical protection. Any available data on antigen-specific T-cell responses including CD4+ T-cells and CD8+ cytotoxic T-lymphocytes and relevant cytokines should be presented taking into account baseline status [7].

Granzyme B, quantified in fresh cell lysates, has been suggested to be a useful marker of cytotoxic T-lymphocyte response and a reliable predictor of influenza illness among the vaccinated elderly population. An influenza-specific GrzB ELISpot assay using cryopreserved peripheral blood mononuclear cells was recently developed and tested in otherwise-healthy elderly patients. No correlation was found between granzyme B activity and hemagglutination inhibition titers, indicating no relationship between the cytolytic activity and humoral antibody levels after influenza vaccination. Additionally, as expected, a significant negative correlation between granzyme B response and age was observed. This possibly suggests that the granzyme ELISpot assay is a useful tool that can be used for the evaluation of influenza vaccine immunogenicity in the elderly, independently of HI [47]. ELISpot also can be used for quantitating interferon-secreting cells, such as IFN-gamma, IL-4, TNF-alpha, and others [47].

Flow cytometry is another possible approach for the characterization of cellular immune responses. Intracellular cytokine staining in protein inhibitor (monensin, Brefeldin A)-treated peripheral mononuclear cells originating from vaccine-treated individuals and a subsequent flow cytometry analysis allow a detailed analysis of cellular immune responses [48]. The profile of the secreted cytokines characterizes the vaccine-induced immune response and T-lymphocyte subpopulations, which participate in the antiviral response [48].

## 6. Live Attenuated Influenza Vaccines (LAIVs)

In addition to the widely used trivalent or quadrivalent inactivated influenza vaccines, the use of LAIVs represents an additional challenge in terms of finding the correlation between immunogenicity and vaccine efficacy. LAIV preparations had poor serum immune responses, especially in direct comparison to TIV [49,50]. Neutralizing antibody titers by MN were higher than HI titers but remained lower than in normal subjects or immunocompromised participants who received TIV [49,50]. Besides HI and MN, with this particular vaccine type, correlates of immune protection should also include secretory immunoglobulin A, although it is much less studied. At this point, with the high efficacy of LAIV vaccines In children up to middle-aged adults, data support the use of this type of vaccine in the age group of 5–49 years [51].

Currently, due to the lack of a convincing correlation between immunogenicity parameters and protection against clinical disease, according to the most recent EMA guideline, seasonal live attenuated influenza vaccines can only be approved based on a demonstration of vaccine efficacy in specific age and other population groups [7]. Additionally, for already authorized LAIVs, subject to prior agreement with competent regulatory authorities, immunological bridging studies may be used to support changes in formulation or delivery devices.

## 7. Summary

For inactivated influenza vaccines containing viral HA, an HI titer of 1:40 was previously suggested to represent a reasonable statistical correlate for an efficacy of 50–70% against clinical symptoms of influenza based on challenge studies in healthy adults. Therefore, most licensing criteria were based on that simple parameter (Table 1). Since then, evidence has emerged to indicate that there remains a need to better define correlates of protection against influenza, which potentially may vary according to individual characteristics, populations, specific age groups (e.g., the pediatric population), and vaccine types.

In summary, although influenza vaccines are among the best studied ones in terms of immunogenicity parameters and correlates of protection, the development of more reliable and reproducible methods is still needed. This is reflected by the changes in the most recent licensing guidelines, shifting the focus from immunogenicity to vaccine efficacy and comparative, non-inferiority trials.

## Figures and Tables

**Table 1:** The immunogenicity requirements for licensing influenza vaccines, as established by the Committee for Human Medicinal Products (CHMP) in 1997. These criteria were in part adopted by the FDA in 2007.

Immunogenicity Criteria	Adult (Age: 18–65 Years)	Elderly (Age: >65 Years)
Proportion of subjects seroconverted * or significant increase in titers.	≥40%	≥30%
Increase in GMT (post/prevaccination ratio) **	>2.5	>2.5
Proportion of subjects seroprotected ***	≥70%	≥60%

* seroconversion meaning either a negative prevaccination serum and a postvaccination serum titer of >1:40 or an increase in the titer postvaccination of at least fourfold. ** GMT: Geometric Mean Titer. The FDA does not use this criterion. *** seroprotection meaning achieving at least a 1:40 titer of hemagglutination inhibition. No separate licensing criteria were established for pediatric patients.
